# Simultaneous photocatalytic degradation and SERS detection of tetracycline with self-sustainable and recyclable ternary PI/TiO_2_/Ag flexible microfibers

**DOI:** 10.1038/s41378-023-00624-x

**Published:** 2024-03-18

**Authors:** Donglai Han, Boyang Guo, Yanru Li, Wei Feng, Keyan Liu, Tianna Wu, Yuchun Wan, Lili Wang, Ming Gao, Yang Liu, Lili Yang, Maobin Wei, Shuo Yang

**Affiliations:** 1 School of Materials Science and Engineering, Changchun University of Science and Technology, 130022 Changchun, China; 2 College of Science, Changchun University, 130022 Changchun, China; 3 Key Laboratory of Functional Materials Physics and Chemistry of the Ministry of Education, Jilin Normal University, 130103 Changchun, China

**Keywords:** Organic-inorganic nanostructures, Structural properties

## Abstract

Facile and efficient photocatalysts using sunlight, as well as fast and sensitive surface-enhanced Raman spectroscopy (SERS) substrates, are urgently needed for practical degradation of tetracycline (TC). To meet these requirements, a new paradigm for PI/TiO_2_/Ag organic‒inorganic ternary flexible microfibers based on semiconducting titanium dioxide (TiO_2_), the noble metal silver (Ag) and the conjugated polymer polyimide (PI) was developed by engineering a simple method. Under sunlight, the photocatalytic characteristics of the PI/TiO_2_/Ag flexible microfibers containing varying amounts of Ag quantum dots (QDs) were evaluated with photocatalytic degradation of TC in aqueous solution. The results demonstrated that the amount of Ag affected the photocatalytic activity. Among the tested samples, PI/TiO_2_/Ag-0.07 (93.1%) exhibited a higher photocatalytic degradation rate than PI/TiO_2_ (25.7%), PI/TiO_2_/Ag-0.05 (77.7%), and PI/TiO_2_/Ag-0.09 (63.3%). This observation and evaluation conducted in the present work strongly indicated a charge transfer mechanism. Moreover, the PI/TiO_2_/Ag-0.07 flexible microfibers exhibited highly sensitive SERS detection, as demonstrated by the observation of the Raman peaks for TC even at an extremely low concentration of 10^–10^ moles per liter. The excellent photocatalytic performance and SERS detection capability of the PI/TiO_2_/Ag flexible microfibers arose from the Schottky barrier formed between Ag and TiO_2_ and also from the outstanding plasmonic resonance and visible light absorptivity of Ag, along with immobilization by the PI. The successful synthesis of PI/TiO_2_/Ag flexible microfibers holds significant promise for sensitive detection and efficient photocatalytic degradation of antibiotics.

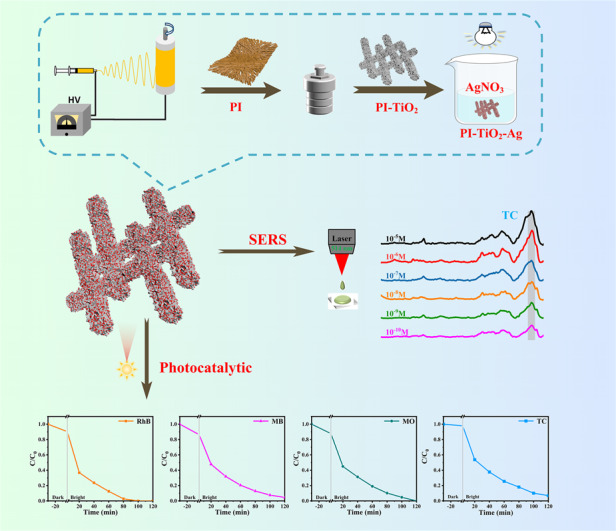

## Introduction

TC has emerged as one of the most extensively employed antibiotics due to its simple synthesis, low cost, excellent antibacterial properties, and broad applicability, and it is used in treating human diseases and as a growth promoter in the livestock and aquaculture industries^1^. However, TC is a continuous antibiotic that is neither fully absorbed by living creatures nor eliminated via traditional water treatment. Untreated TC seriously impairs human health and the ecological balance^2^, so finding a method for effective detection and elimination of TC residues is crucial.

SERS is a spectroscopic detection technique that allows simple, rapid, and sensitive determination of the spectra of target analytes based on interactions between Raman substrates and target analytes during illumination^3^. Photocatalysis is a green technology that relies on the ability of photocatalysts to decompose pollutants under irradiation without generating resource waste^4^. TiO_2_ is one of the most extensively studied materials in the field of photocatalysis, primarily due to its nontoxicity, cost-effectiveness, high efficiency, high oxidation capacity, photostability and relatively high chemical stability^5^. Even more surprising is that TiO_2_ exhibits high Raman activity^6^. However, the wide bandgap and easy recombination of charge carriers in TiO_2_ limit its use in photocatalysis^7^, and the chemical enhancement mechanism of Raman is not sufficient to meet the requirements of sensitive detection^6^. To overcome these drawbacks of TiO_2_, heterojunctions composed of TiO_2_ and other noble metals have been investigated, for example, by combining TiO_2_ with gold, silver, nickel, or platinum^8–10^. Among various noble metals, Ag was used to form a composite material with TiO_2_ due to its strong surface plasmon resonance (SPR) absorption and significant charge capture in the visible spectrum^11,12^. This approach utilizes the visible light absorptivity of Ag to extend the absorption range and enhance the efficiency of sunlight utilization and maximizes the use of Ag plasmonic resonance and the Schottky barrier formed at the Ag-TiO_2_ interface to inhibit recombination of the photogenerated charge carriers and enhance charge resonance transfer^13^. Consequently, it significantly enhances photocatalytic degradation and SERS detection. However, it should be noted that TiO_2_/Ag composite materials are mostly powders, which can easily cause secondary pollution and are not conducive to subsequent recycling^11,12^. The most common approaches used to address these issues involve loading the powdered samples onto magnetic carriers, ITO, and silicon dioxide^14–16^. Beyond that, organic flexible nanofibers prepared by electrospinning have also served as carriers to immobilize TiO_2_/Ag powders, enhance recyclability and prevent secondary pollution^17^. PI is one of the most commonly utilized organic flexible nanofibers, owing to its remarkable flexibility, large specific surface area and high porosity^18^. The incorporation of TiO_2_/Ag into PI would enhance the dispersion of TiO_2_/Ag and improve its photocatalytic and SERS performance while enabling recycling and mitigating the risk of secondary pollution^19^.

In the present work, we developed a facile approach for the fabrication of PI/TiO_2_/Ag organic‒inorganic ternary flexible microfibers exhibiting sensitive SERS detection and efficient photocatalytic degradation of TC under simulated sunlight. The photocatalytic capabilities of the PI/TiO_2_ and PI/TiO_2_/Ag flexible microfibers were assessed under simulated solar light via degradation of TC as a model compound. The SERS detection efficiency of the PI/TiO_2_/Ag-0.07 flexible microfibers was evaluated with 4-aminophenol (4-ATP) and TC. The structural characteristics of the PI/TiO_2_/Ag flexible microfibers and the role of the Ag QDs in enhancing the photocatalytic and SERS activity of PI/TiO_2_ are the focus of the present study and discussion.

## Results and discussion

### Structural and morphological characterization

The structural and morphological features of PI, PI/TiO_2_ and the PI/TiO_2_/Ag flexible nano/microfibers were studied with several analytical techniques, including Fourier transform infrared spectroscopy (FTIR), thermogravimetric analysis (TGA), X-ray diffraction (XRD), energy dispersive X-ray spectroscopy (EDX), scanning electron microscopy (SEM), transmission electron microscopy (TEM) and X-ray photoelectron spectroscopy (XPS).

**Fig. 1:**
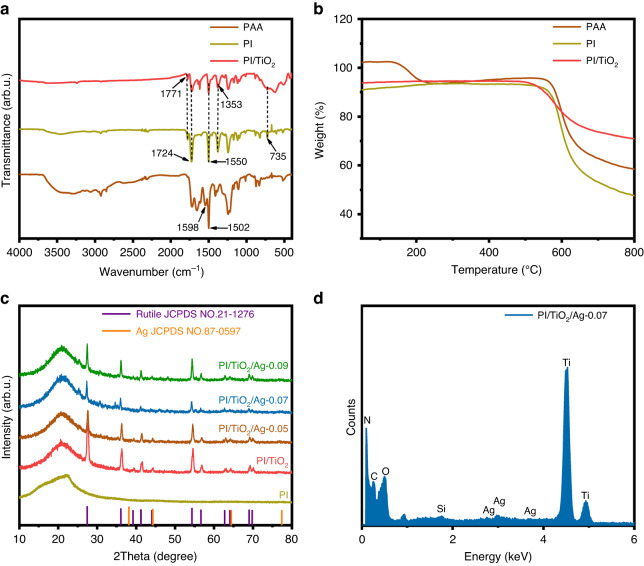
Structural characteristics and thermal stability of flexible fibers. **a** FTIR spectra and **b** TGA curves for PAA, PI and the PI/TiO_2_ flexible fibers. **c** XRD patterns for PI, PI/TiO_2_ and the PI/TiO_2_/Ag flexible fibers. **d** EDX spectrum of the PI/TiO_2_/Ag-0.07 flexible microfibers

FT-IR spectroscopy was used to determine structures and chemical bonds of the polyamide acid (PAA), PI, and PI/TiO_2_ flexible fibers. As exhibited in Fig. 1a, the absorption peaks at 1598 and 1502 cm^−1^ were attributed to the C = O stretching vibration and N–H vibration of PAA^20^. The absorption peaks at 1771, 1724, 1550, 1353, and 735 cm^−1^ were associated with asymmetric stretching and symmetric stretching vibrations of aromatic C = N bonds in the triazine unit, stretching vibrations of C–N–C moieties in the five-membered imide rings and bending vibrations of the C = O bonds in PI, respectively^21^. The broad peaks at 400 to 800 cm^−1^ represented Ti–O–Ti stretching vibrations, which confirmed in situ surface growth of TiO_2_ on the PI^22^. These findings indicate that the primary chemical structure and bonding of the PI remained unaffected by TiO_2_ deposition.

The thermal stabilities were investigated with TGA. As shown in Fig. 1b, the imidization reaction of PAA began at approximately 200 °C. As the temperature reached 300 °C, the macromolecular chains within the PAA nanofibers underwent rearrangement to a more stable conformation and ultimately formed PI nanofibers. Based on the thermal weight loss curves for PAA and the PI nanofibers at 300 °C, the PI nanofibers no longer exhibited thermal weight losses, and hence, there was no imidization reaction. From that, we inferred that after 2 h of thermal imidization of the PAA nanofibers at 300 °C, the internal molecular chains of PI adopted stable conformations and showed good thermal stability. At approximately 580 °C, an obvious inflection appeared because the molecular chains of the PI nanofibers began to break. At this point, the thermal weight loss rate reached a maximum due to carbonization of the PI nanofibers. At approximately 800 °C, they are almost fully converted into carbon fibers^19^. Notably, the weight loss from PI/TiO_2_ is less than that from PI, which was ascribed to the addition of TiO_2_. These results showed that our prepared PI and PI/TiO_2_ nanofibers were thermally stable.

The generated samples were subjected to XRD analyses to investigate their crystal structures and phase transitions. Figure 1c depicts the XRD patterns for PI, PI/TiO_2_ and the PI/TiO_2_/Ag flexible fibers. The prominent peak at 2θ = 27.9° was related to the interlayer π-π stacking of PI, specifically corresponding to the (001) facet of PI^23^. The primary diffraction peaks for PI/TiO_2_ and PI/TiO_2_/Ag were comparable. The typical diffraction peaks at 27.4°, 36.1°, 39.2°, 41.2°, 44.1°, 54.3°, 56.6°, 62.7°, 64.0°, 69.0° and 69.8° were attributed to crystalline rutile TiO_2_ (JCPDS NO. 21-1276)^24^. However, oddly, the XRD patterns for PI/TiO_2_/Ag exhibited no diffraction peaks for Ag. This may be explained by the low amount of Ag present or by the fact that the diffraction peaks for the Ag QDs were obscured by those of TiO_2_. Nevertheless, the EDX spectrum of PI/TiO_2_/Ag-0.07 confirmed the presence of Ag. As shown in Fig. 1d, Ag, Ti, O, C and N were detected. The aforementioned results demonstrated the formation of PI/TiO_2_/Ag.

**Fig. 2:**
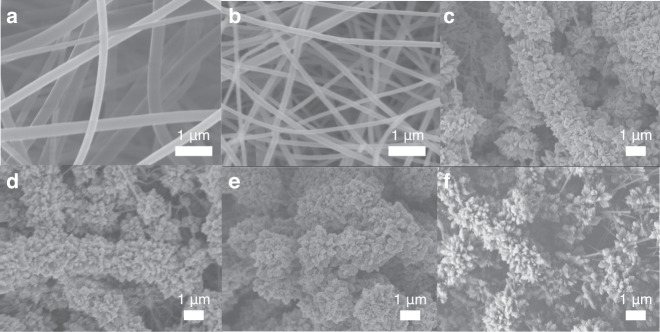
Surface morphology of flexible fibers. SEM images of **a** PAA, **b** PI, **c** PI/TiO_2_, **d** PI/TiO_2_/Ag-0.05, **e** PI/TiO_2_/Ag-0.07 and **f** PI/TiO_2_/Ag-0.09 flexible fibers

The morphologies of PAA, PI, PI/TiO_2_ and PI/TiO_2_/Ag were examined with SEM. As illustrated in Fig. 2a, b, the PAA and PI nanofibers exhibited smooth and uniform surfaces. However, the diameters of the PI nanofibers (approximately 200 nm) were smaller than those of the PAA nanofibers (approximately 250 nm). This was due to volatilization of the DMF solvent and the thermal imidization reactions occurring during calcination. As seen from Fig. 2c, TiO_2_ nanorods (NRs) with petal-like shapes were uniformly grown on the surfaces of the PI nanofibers. However, the Ag QDs were not readily visible in Fig. 2d–f.

**Fig. 3:**
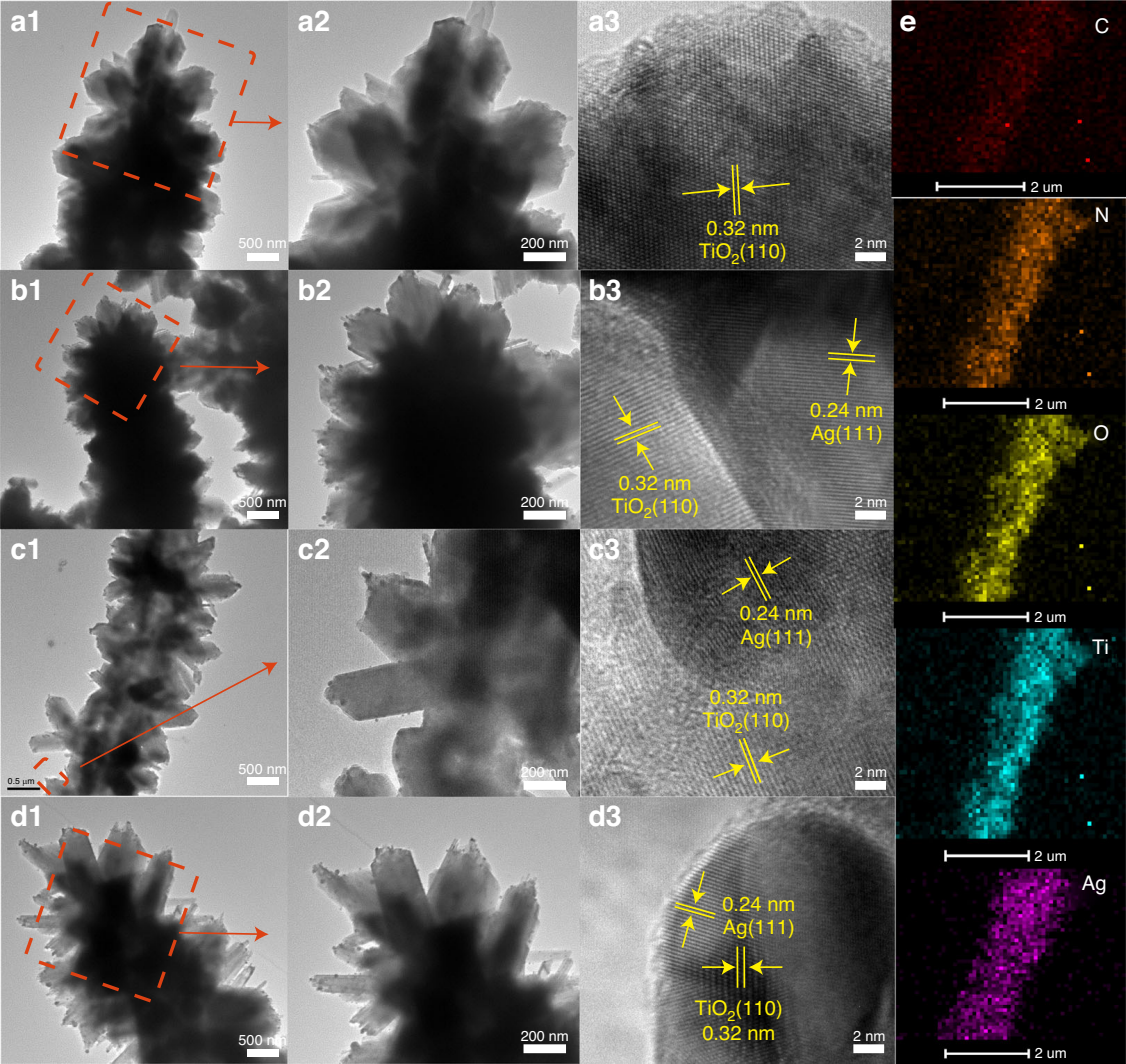
Microstructure and element distribution of flexible fibers. TEM and HRTEM images of **a** PI/TiO_2_, **b** PI/TiO_2_/Ag-0.05, **c** PI/TiO_2_/Ag-0.07 and **d** PI/TiO_2_/Ag-0.09 flexible microfibers. **e** EDX element maps for C, N, O, Ti and Ag in the PI/TiO_2_/Ag-0.07 flexible microfibers

Furthermore, the microstructures of PI/TiO_2_ and PI/TiO_2_/Ag were investigated with TEM and high-resolution TEM (HRTEM). Figure 3(a1–d1) confirms that in PI/TiO_2_, TiO_2_ nanorods (size: approximately 250 nm) were uniformly and compactly grown on the surfaces of the PI nanofibers. Figure 3a2–d2 also shows that the number of Ag DQs on the surface of PI/TiO_2_ gradually increased with increasing concentrations of AgNO_3_. Figure 3a3–d3 demonstrates that Ag DQs were bonded to PI/TiO_2_ and exhibited an average size of 15 ± 5 nm, and the PI/TiO_2_/Ag samples included both TiO_2_ NR and Ag DQ crystals. The lattice spacing of 0.32 nm observed in the figure matched that of the (110) crystal plane of TiO_2_ (JCPDS NO. 21-1276)^25^. The lattice distance of 0.24 nm in the figure corresponded to the (111) crystal plane of Ag (JCPDS NO. 87-0597)^26^. The elemental maps for the PI/TiO_2_/Ag-0.07 microfibers are depicted in Fig. 3e, which indicates that the C, O, N, Ti and Ag elements were well distributed throughout the PI/TiO_2_ nanofibers. Additionally, the distribution diameters of C and N in PI were smaller than that of Ti in TiO_2_, and the distribution diameter of Ti in TiO_2_ was smaller than that of Ag, indicating the formation of a hierarchical core-shell structure.

**Fig. 4:**
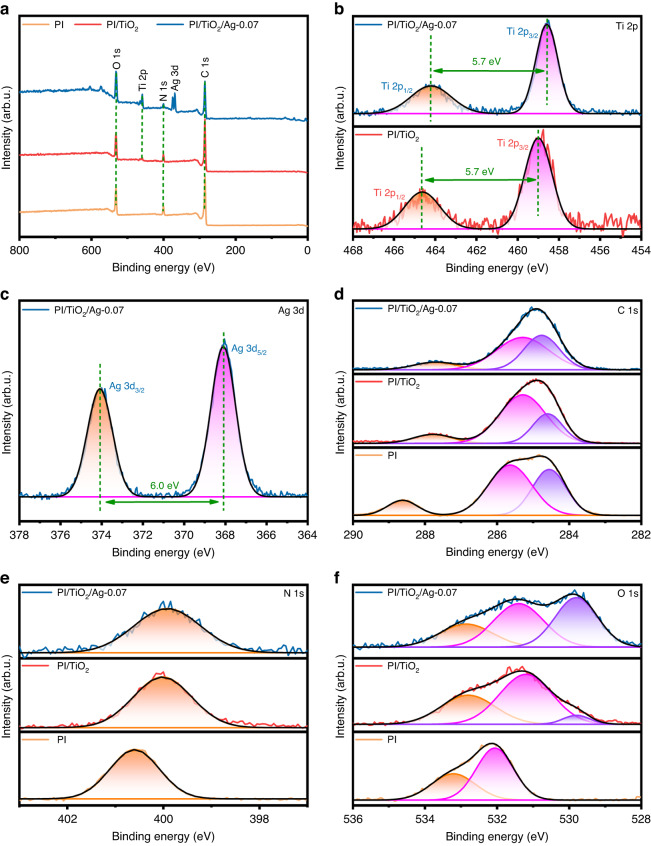
Electronic states and chemical compositions of lexible fibers. XPS data for PI, PI/TiO_2_ and the PI/TiO_2_/Ag-0.07 flexible fibers: **a** full survey; **b** Ti 2p; **c** Ag 3d; **d** C 1s; **e** N 1s and **f** O 1s XPS data

Furthermore, the valence states and elemental compositions of the prepared samples were verified with XPS analyses. As observed in Fig. 4a, the peaks for C, N and O in PI/TiO_2_ and PI/TiO_2_/Ag were consistent with those for PI. The peaks observed in Fig. 4b with binding energies of 458.5 eV and 464.2 eV were assigned to Ti 2p_3/2_ and Ti 2p_1/2_, and the 5.7 eV splitting between them indicated the presence of Ti^4+^^27^. The Ag 3d XPS spectrum of PI/TiO_2_/Ag-0.07 is shown in Fig. 4c. The peaks at 368.7 eV and 374.7 eV indicated the 3d_5/2_ and 3d_3/2_ states of metallic Ag (0), respectively. Notably, the energy difference of 6.0 eV between the Ag 3d_3/2_ and Ag 3d_5/2_ binding energies was characteristic of metallic Ag^28^. Figure 4d displays the C 1 s XPS data for PI, PI/TiO_2_, and PI/TiO_2_/Ag-0.07, which contained three peaks located at 288.2 eV, 285.8 eV, and 284.6 eV, respectively. The binding energies of these peaks were calibrated relative to the C 1s peak at 284.6 eV, which was assigned to carbon contamination or *sp*^*2*^ C-C/C = C bonds. In comparison, the peaks at 288.3 eV and 285.8 eV were attributed to the O–C = O bonds in the triazine rings and the C–N bonds in PI, respectively^29^. As shown in Fig. 4e, the N 1 s peak at 400.4 eV was associated with the N–(C)_3_ bonds of PI^30^. In Fig. 4f, the O 1s spectrum of PI displayed two peaks at 532.0 and 533.2 eV, which were attributed to C = O and C–O or adsorbed water, respectively. In comparison, PI/TiO_2_ and PI/TiO_2_/Ag showed one additional peak at 529.8 eV, which corresponded to the Ti–O bonds^31,32^. Notably, in Fig. 4d–f, the peaks for PI/TiO_2_ were redshifted relative to those for PI, and the peaks for PI/TiO_2_/Ag exhibited redshifts relative to those for PI/TiO_2_, indicating successful incorporation of TiO_2_ into PI, as well as PI, TiO_2_, and Ag. In addition, the Ti 2p and O 1s binding energies for PI/TiO_2_/Ag were redshifted toward lower energy compared to those for PI/TiO_2_, indicating electron transfer from Ag to TiO_2_.

### Photodegradation of TC

**Fig. 5:**
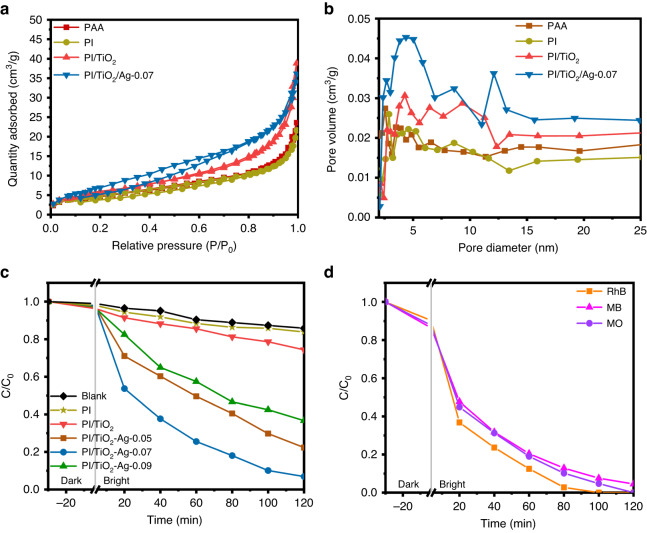
Photocatalytic activity of flexible fibers. **a** Nitrogen adsorption-desorption isotherms and **b** pore size distributions for PAA, PI, PI/TiO_2_ and the PI/TiO_2_/Ag flexible fibers. **c** Photodegradation curves for TC and **d** curves for Rh B, MB and MO photodegradation with the PI/TiO_2_/Ag-0.07 flexible microfibers under simulated sunlight

To ascertain the specific surface areas and pore structures of the synthesized photocatalysts, nitrogen adsorption-desorption isotherms were generated. As shown in Fig. 5a, the isotherms for PAA, PI, PI/TiO_2_ and the PI/TiO_2_/Ag flexible fibers were all type IV isotherms with type H3 hysteresis loops, implying that slit-like mesoporous structures were formed. The specific surface areas for PAA, PI, PI/TiO_2_ and the PI/TiO_2_/Ag flexible fibers were estimated to be 17, 17, 21, and 29 m^2^/g, respectively. Clearly, the PI/TiO_2_/Ag flexible microfibers had the highest specific surface area. Figure 5b shows that a significant proportion of the pores had diameters ranging from 0 to 15 nm, which is consistent with the SEM and TEM images. These abundant active sites in the hierarchical mesoporous nanostructure are believed to result in the high photocatalytic activity and facile photocatalytic reactions^33^.

As seen in Fig. 5c, the photocatalytic capabilities of PI, PI/TiO_2_ and the PI/TiO_2_/Ag flexible fibers with different amounts of Ag QD were investigated with photocatalytic degradation of TC under simulated sunlight. PI and PI/TiO_2_ exhibited very poor catalytic performance in degrading TC (16.2% and 25.7% conversions after 120 min) due to inefficient photogenerated carrier separation and transfer. Then, we loaded Ag QDs on the PI/TiO_2_ microfibers. As the content of AgNO_3_ was increased from 0.05 M to 0.07 M, the photocatalytic efficiency for TC degradation increased from 77.7% to 93.1%. However, as the concentration of AgNO_3_ was increased to 0.09 M, the photocatalytic degradation efficiency did not continue to increase but rather decreased to 63.3%. The enhanced photocatalytic efficiency was attributed to multiple factors^26^. First, the formation of a Schottky barrier between Ag and TiO_2_ provided efficient separation of the photogenerated electrons and holes. Additionally, the remarkable SPR and visible light absorptivity of Ag enhanced the photocatalytic activity and solar energy utilization efficiency. Moreover, the immobilization capability of PI and incorporation of the Ag QDs resulted in a higher specific surface area, which also enhanced the photocatalytic efficiency. However, when more Ag QDs (0.09 M AgNO_3_) were loaded, the excess Ag QDs tended to occupy some of the reactive sites, which inhibited the formation of photocatalytic intermediates and the adsorption of TC, and consequently reduced the photocatalytic performance^34,35^. Additionally, PI/TiO_2_/Ag was employed for degradation of Rhodamine B (Rh B), Methylene Blue (MB) and Methyl Orange (MO). As shown in Fig. 5d, PI/TiO_2_/Ag exhibited excellent photocatalytic degradation activity with these three pollutants (100%, 95.5%, and 100%), confirming its high efficiency as a versatile photocatalyst suitable for the removal of diverse pollutants.

### Photoreduction mechanism of TC

**Fig. 6:**
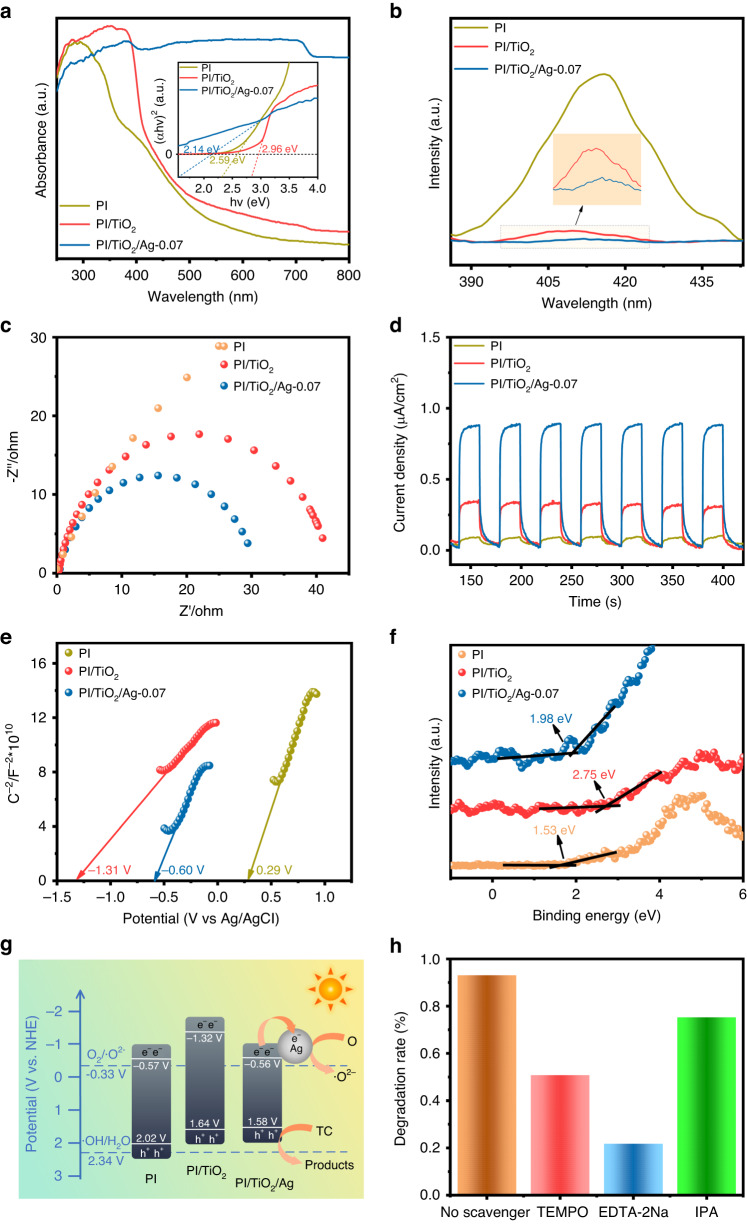
Optical properties, electrochemical performence and photocatalytic machanism of flexible fibers. **a** Ultraviolet‒visible diffuse reflectance spectra and Tauc plots, **b** PL, **c** EIS and **d** transient photocurrent response spectra, **e** Mott–Schottky curves, **f** VB-XPS spectra, **g** energetics for photocatalytic degradation of TC by PI, PI/TiO_2_ and the PI/TiO_2_/Ag-0.07 flexible fibers and **h** effects of different scavengers on the photocatalytic degradation of TC by the PI/TiO_2_/Ag-0.07 flexible microfibers

The absorptivities and band gaps (*E*_g_) of PI, PI/TiO_2_ and the PI/TiO_2_/Ag-0.07 flexible fibers were determined with ultraviolet‒visible diffuse reflectance spectroscopy (UV‒vis DRS) and the corresponding Tauc plots. In Fig. 6a, the absorption spectra of PI and PI/TiO_2_ exhibited significant peaks in the UV region, and they decreased rapidly in the visible region. When the Ag QDs were deposited, as shown in the UV‒vis DR spectrum of PI/TiO_2_/Ag-0.07, absorption of visible wavelengths was greatly enhanced, which was attributed to SPR absorption by the Ag QDs^36^. The inserted plots in Fig. 6a depict the Tauc curves for PI, PI/TiO_2_ and PI/TiO_2_/Ag-0.07. The graphs show that the *E*_g_ values were 2.59 eV, 2.96 eV and 2.14 eV.

The photocatalytic activity and separation of the photogenerated charge carriers in the obtained samples were evaluated with the photoluminescence (PL) spectra generated with an excitation wavelength of 350 nm and electrochemical impedance spectroscopy (EIS). As shown in Fig. 6b, PI exhibited a prominent peak in the wavelength range 400–500 nm. Similar peaks for PI/TiO_2_ and PI/TiO_2_/Ag-0.07 were centered at approximately 410 nm. This was attributed to the fact that the Ag 3d orbitals accepted electrons and captured the charge carriers to inhibit recombination of the photogenerated electrons and holes^37,38^. Noticeably, the PL intensity of PI/TiO_2_/Ag-0.07 was much lower than that of PI/TiO_2_, suggesting a reduced rate for electron-hole recombination and improved photocatalytic activity. In other words, the synergy of the Ag QDs and the TiO_2_ NR interlayer facilitated transport of the photogenerated charge carriers. Here, we also note that charge transfer increased the photocatalytic efficiency of PI/TiO_2_/Ag and also improved its SERS performance^39^. Figure 6c also shows Nyquist plots for various flexible nano/microfibers. The radius of the arc reflects the reaction rate at the surface of the flexible composite fiber, which can be interpreted as the charge transfer resistance (Rct). It was discovered that PI/TiO_2_/Ag-0.07 has a much smaller Rct than PI/TiO_2_ and PI, revealing that the PI/TiO_2_/Ag-0.07 flexible microfibers showed the highest efficiency in separating the photogenerated electron-hole pairs and facilitating rapid interfacial charge transfer^40^.

Furthermore, photocurrent response (i-t) experiments were performed under simulated sunlight with on/off cycles of 20 s. As illustrated in Fig. 6d, the as-prepared samples exhibited fairly stable photocurrent responses. In particular, the PI/TiO_2_/Ag-0.07 flexible microfibers showed much higher photocurrents and transient photocurrent densities than PI/TiO_2_ and PI, indicating that PI/TiO_2_/Ag-0.07 separated the photogenerated electrons most effectively. This enhanced performance was attributed to effective interfacial charge transfer facilitated by the tight bonding and synergy of the TiO_2_ NRs and Ag QDs. Furthermore, this unique combination contributed to the excellent photocatalytic and SERS activities of PI/TiO_2_/Ag-0.07.

The semiconducting and flat band (Fermi energy level, *E*_F_) potentials were determined via electrochemical Mott-Schottky (MS) tests. Figure 6e illustrates the potentials for PI, PI/TiO_2_ and PI/TiO_2_/Ag-0.07, which were 0.29 V, −1.31 V and −0.60 V (vs. Ag/AgCl), respectively. These values were referenced to the normal hydrogen electrode (NHE) potential with the following formula:

1 $${E}_{{\rm{NHE}}}={E}_{{{\rm{Ag}}}/{{\rm{Ag}}}{\rm{Cl}}}+0.198{\rm{V}}$$

Thus, the calculated *E*_F_ potentials for PI, PI/TiO_2_ and PI/TiO_2_/Ag-0.07 were 0.49 V, −1.11 V and −0.40 V vs. NHE, respectively. Furthermore, the VB-XPS spectra (Fig. 6f) were used to determine the relative potentials of the valence bands (VB) vs. the Fermi energy level, and the estimated values for PI, PI/TiO_2_, and PI/TiO_2_/Ag-0.07 were 1.53 eV, 2.75 eV, and 1.98 eV, respectively. The VB potentials for PI, PI/TiO_2_ and PI/TiO_2_/Ag-0.07 were calculated as 2.02 V, 1.64 V and 1.58 V, and the conduction band (CB) potentials were −0.57 V, −1.32 V and −0.56 V. Based on the previously described band structures, a mechanism for photocatalytic degradation of TC by PI/TiO_2_/Ag is proposed in Fig. 6g. Under simulated sunlight, the Ag QDs generated an intense electric field near their plasmon frequency, which excited the electrons of TiO_2_ from the VB to the CB^41^. Subsequently, electron transfer from the CB of TiO_2_ to Ag converted O_2_ into ·O^2-^ (the superoxide anion radical) (−0.33 V vs. NHE). Nonetheless, the holes generated in the valence band of TiO_2_ did not convert H_2_O into ·OH (hydroxyl free radical) due to the negative potential of the TiO_2_ valence band compared to the potential of ·OH/H_2_O (2.34 V vs. NHE)^42,43^. Two protons, an e^−^ and ·O^2−^ reacted to produce a small amount of ·OH. Importantly, the energy levels for Ag and the CB of TiO_2_ were different. As a result, electrons migrated from the CB of TiO_2_ to the Fermi level of Ag, which maintained electron/hole separation and accelerated the deterioration of TC^44^. The photocatalytic process can be described with the following equations:

2 $${\rm{PI}}/{{\rm{TiO}}}_{2}/{\rm{Ag}}+{hv}\to {{\rm{TiO}}}_{2}({{\rm{h}}}^{+})+{\rm{Ag}}({{\rm{e}}}^{-})$$

3 $${{\rm{O}}}_{2}+{{\rm{e}}}^{-}\to {{{\cdot {\rm{O}}}}}^{2-}$$

4 $${{{\cdot {\rm{O}}}}}^{2-}+{{\rm{e}}}^{-}+{2{\rm{H}}}^{+}\to {{\rm{H}}}_{2}{{\rm{O}}}_{2}$$

5 $${{\rm{H}}}_{2}{{\rm{O}}}_{2}+{{\rm{e}}}^{-}\to {\rm{\cdot OH}}+{{\rm{OH}}}^{-}$$

6 $${{{\cdot {\rm{O}}}}}^{2-}/{{{\cdot {\rm{OH}}}}/{\rm{h}}}^{+}+{\rm{TC}}\to {\rm{Products}}$$

To provide intuitive verification of the active species involved in the photocatalytic mechanism described above, the efficiency of TC degradation by PI/TiO_2_/Ag-0.07 was investigated with various scavengers. As displayed in Fig. 6h, the efficiency of TC degradation was reduced by the addition of different reactive scavengers. Specifically, the addition of 1 mM EDTA-2Na^45^ and 4-hydroxy-TEMPO^46^ as scavengers for h^+^ and ·O^2−^, respectively, resulted in substantial decreases in the rate of TC degradation. Only 21.8% and 26.8% of the TC was degraded, which were much lower than the degradation rates seen without scavengers (93.1%). This strong inhibition implied that both h^+^ and ·O^2−^ were important reactive species, although h^+^ had a slightly greater influence. Moreover, in using 1 mM IPA^47^ as a scavenger for ·OH, the photocatalytic rate of TC degradation was reduced from 93.1% to 75.3%, indicating the significance of ·OH in the degradation of TC. These results were consistent with the photocatalytic mechanism.

### SERS activity

**Fig. 7:**
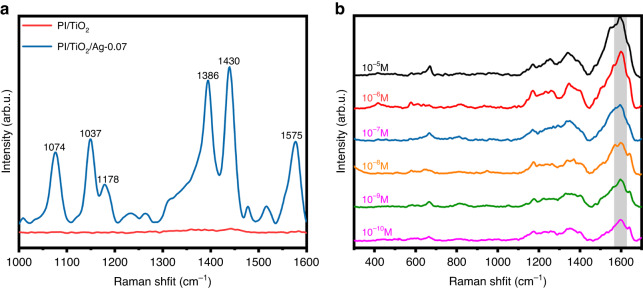
SERS detection sensitivity of PI/TiO_2_/Ag-0.07 flexible fibers. **a** SERS spectra of 4-ATP adsorbed on PI/TiO_2_ and PI/TiO_2_/Ag-0.07 substrates from a 1 × 10^−5^ M aqueous solution. **b** SERS spectra of the PI/TiO_2_/Ag-0.07 substrate incubated with TC solutions at various concentrations

As noted above, PI/TiO_2_/Ag catalyzed photocatalytic degradation and enhanced SERS detection of the pollutants. As SERS substrates, PI/TiO_2_ and PI/TiO_2_/Ag-0.07 were utilized to detect 4-ATP in a 1 × 10^–5 ^M solution to identify the better SERS substrate. As shown Fig. 7a, the peaks at 1137 cm^−1^, 1386 cm^−1^, and 1430 cm^−1^ corresponded to the “b2” vibrational modes v_C-C_ + v_C-S_, β_C-H_, and v_C-C_, respectively. Additionally, the peaks at 1074 cm^−1^, 1178 cm^−1^, and 1575 cm^−1^ represented the “a1” vibration modes attributed to v_C-C_ + v_C-S_, β_C-H_, and v_C-C_, respectively^48^. The peak intensity for 4-ATP enhanced with PI/TiO_2_ was very weak. However, when PI/TiO_2_/Ag-0.07 was applied, the intensity of the SERS signal was significantly enhanced, which indicated that PI/TiO_2_/Ag enabled sensitive detection of 4-ATP. The enhancement originated from two factors: first, under irradiation, localized surface plasmon resonance (LSPR) of the Ag QDs formed a local electromagnetic field and accelerated charge transfer in PI/TiO_2_/Ag; second, the Schottky barrier formed by the Ag QDs also facilitated charge transfer. The efficient charge transfer enhanced the SERS signal^49,50^. We then used the PI/TiO_2_/Ag-0.07 substrate for SERS detection of TC. As shown in Fig. 7b, the SERS signal decreased gradually as the TC concentration was decreased, but even at a low concentration of 1 × 10^–10 ^M, the PI/TiO_2_/Ag-0.07 substrate still generated an appreciable SERS signal.

## Conclusions

In summary, we employed a simple and effective strategy to fabricate novel PI/TiO_2_/Ag organic‒inorganic ternary flexible composite microfibers that exhibited both sensitive SERS detection and highly efficient photocatalytic degradation. The proportion of Ag QDs was a key factor influencing the photocatalytic and SERS performance of the fabricated samples. As the content of Ag was increased, the efficiency for photocatalytic degradation with PI/TiO_2_/Ag initially increased and then decreased. The initial increase was attributed to the combined contributions of SPR and visible light absorption by Ag, as well as the Schottky barrier formed between Ag and TiO_2_. The decreased photocatalytic degradation performance was ascribed to the excess Ag occupying the reactive sites. A photocatalytic degradation mechanism for PI/TiO_2_/Ag was proposed and validated with free radical scavenging experiments. Among the samples, the PI/TiO_2_/Ag-0.07 flexible microfibers exhibited the best photocatalytic performance, achieving degradation efficiencies of 93.1%, 95.5%, 100% and 100% for TC, MB, MO and Rh B, respectively. Additionally, the PI/TiO_2_/Ag-0.07 flexible microfibers enabled sensitive SERS detection, as evidenced by the peaks observed even at a low TC concentration of 10^–10 ^M. It is believed that these new perspectives for the design of innovative, highly efficient, and multifunctional organic‒inorganic ternary flexible composite microfibers will contribute to advancements in energy conservation and environmental protection.

## Experimental section

### Materials

No additional purification was carried out on any of the materials before they were used. Pyromellitic dianhydride (PMDA), 4,4′-oxydianiline (4,4′-ODA), titanium, ascorbic acid, a solution of ethylenediaminetetraacetic acid (EDTA-2Na) and 4-hydroxy-TEMPO were purchased from Aladdin. AgNO_3_ and hydrochloric acid solution (HCl) were obtained from Beijing Chemical Works. N,N-Dimethylformamide (DMF), isopropyl alcohol (IPA) and TC were purchased from Macklin.

### Synthesis of PI nanofibers

4,4′-ODA (0.4 g) was added to 3.3 mL of DMF and dissolved by continuous stirring for 30 min. In an ice-water bath, 0.433 g of PMDA was added to the mixed solution in batches and vigorously stirred for 12 h. Subsequently, a light-yellow viscous solution (PAA) was obtained. The combined solution was then poured into a plastic syringe with a 3 mL capacity for electrospinning. Aluminum foil served as the collector, while a 12 kV electrical potential was applied with a 15 cm electrode spacing. Afterward, the PAA nanofibers were removed and placed into a muffle furnace, where they were heated to 300 °C at a rate of 2.5 °C/minute and maintained at this temperature for 2 h. Finally, the PI nanofibers were successfully prepared.

### Synthesis of the PI/TiO_2_ composite

To a vessel with a polytetrafluoroethylene liner, 0.045 g of titanium, 3 mL of 12 M HCl, and 33 mL of deionized water were added. Then, the PI nanofibers were soaked in this solution for 2 h. The autoclave vessel was put in the oven and maintained at 160 °C for 14 h. The obtained sample was repeatedly washed with deionized water and then dried in a 60 °C oven. The TiO_2_ nanorod array was successfully “planted” on top of the brilliant yellow PI nanofiber membrane, and the PI/TiO_2_ composite microfibers were created, as was evident from the layer of silver-gray that formed on the surface.

### Synthesis of the PI/TiO_2_/Ag composite

**Scheme 1:**
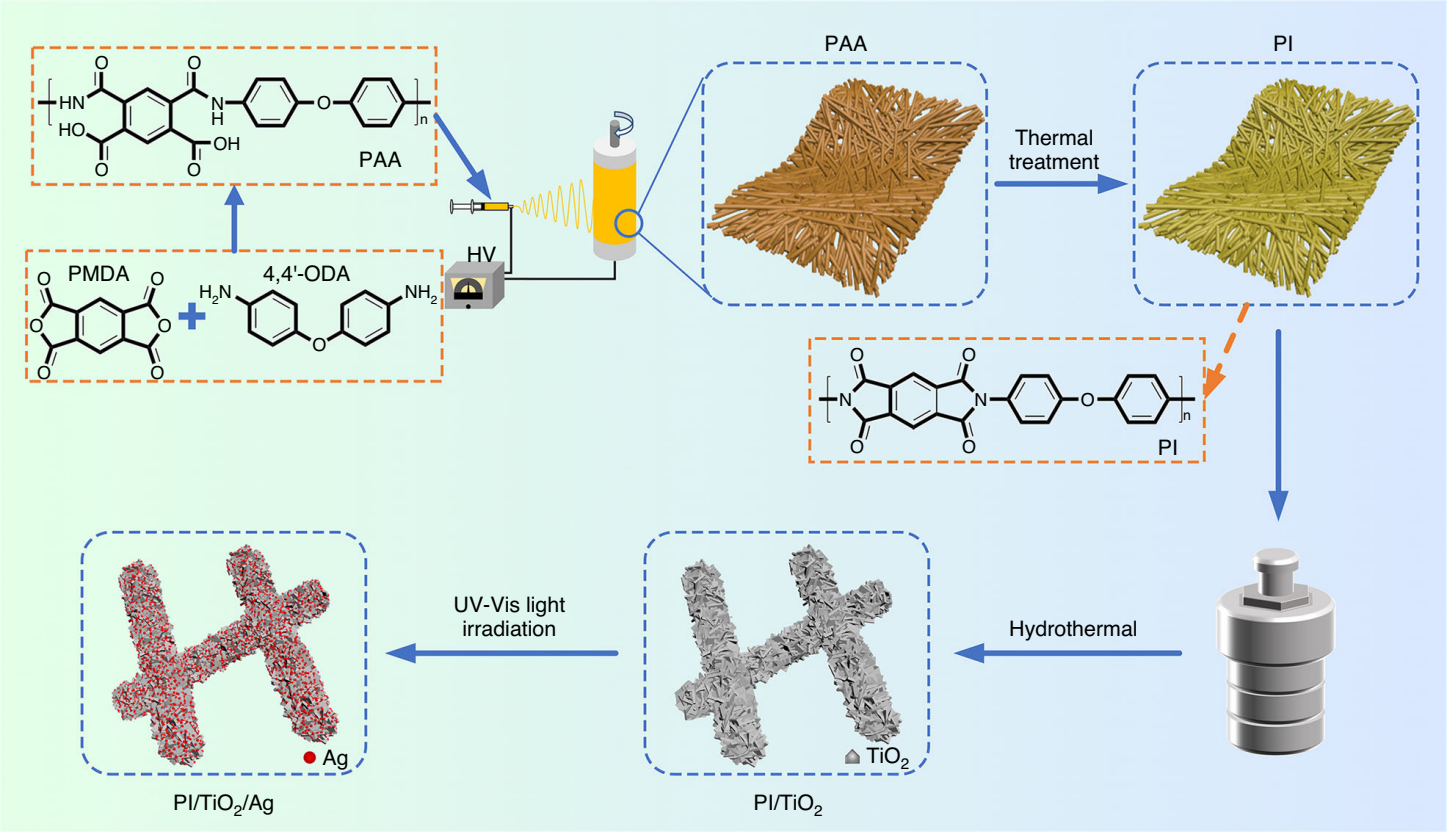
Preparation process of PI/TiO /Ag flexible fibers. Synthetic scheme for the PI/TiO_2_/Ag flexible microfibers

Ternary PI/TiO_2_/Ag composite microfibers were created with electrospinning, a hydrothermal approach, and a photoreduction method (as shown in Scheme 1). The PI/TiO_2_ composite nanofibers were irradiated with simulated sunlight from an Xe lamp for 1 h. Then, the PI/TiO_2_ composite microfibers were immersed in 0.05, 0.07 and 0.09 M AgNO_3_ solutions for 4 h. After drying under vacuum, the microfibers were again irradiated for 2 h to create the PI/TiO_2_/Ag composite microfibers. The samples created with the various concentrations were designated PI/TiO_2_/Ag-0.05, PI/TiO_2_/Ag-0.07, and PI/TiO_2_/Ag-0.09.

### Characterization

KBr pellets were used to examine the FTIR spectra with a SHIMADZU FTIR-8400s spectrophotometer. A Bruker AXS D2 PHASER was employed to record XRD patterns with Cu Kα radiation. A NETZSCH STA 449F3 system was used to conduct TGA studies under air, with a heating rate of 10 °C/min up to 800 °C. SEM with a JSM-6701F system and TEM with a Tecnai F20 system equipped with EDX were used to analyze the morphologies and elemental distributions of the photocatalysts. A Micrometrics ASAP-2460 Surface Area and Porosimetry Analyzer was used to measure the BET-specific surface areas. A Thermo Fisher ESCALAB 250Xi system was used to obtain the XPS data. The PL spectra were obtained at room temperature with a Hitachi F-4700 spectrophotometer with an excitation wavelength of 350 nm. A CS310H electrochemical workstation was used to assess the electrochemical performance.

### Photocatalytic degradation of TC

The rate of TC degradation under simulated sunlight was measured to assess the photocatalytic activities of PI, PI/TiO_2_, and PI/TiO_2_/Ag. The experimental process was as follows: to achieve adsorption-desorption equilibrium, a piece of the photocatalyst was soaked in a TC solution (5 mg/L) and maintained in darkness for 30 min. At 20-min intervals, the concentrations of TC were detected at 355 nm with a SHIMADZU UVmini-1240 spectrophotometer.

### SERS measurements

The wavelength, power, exposure time and attenuation degree of the laser used for the SERS measurement were 514 nm, 15 mW/cm^2^, 15 s, and 10%, respectively. For the detection of 4-ATP and TC (solution state), analyte solutions (in EtOH) of various concentrations were applied to mesoporous PI/TiO_2_ and PI/TiO_2_/Ag microfibers by drip-casting. After the sample was blown to a semidry state with an ear washing ball, the SERS signal was measured.

## References

[1] Hunge YM, Yadav AA, Kang S-W, Kim H (2022). Photocatalytic degradation of tetracycline antibiotics using hydrothermally synthesized two-dimensional molybdenum disulfide/titanium dioxide composites. J. Colloid Interface Sci.

[2] Liu L (2021). Treatment of industrial dye wastewater and pharmaceutical residue wastewater by advanced oxidation processes and its combination with nanocatalysts: a review. J. Water Process..

[3] Ying, Y., Tang, Z. & Liu, Y. Material design, development, and trend for surface-enhanced Raman scattering substrates. *Nanoscale* **15**, 10860–10881 (2023). 10.1039/d3nr01456h 37335252

[4] Amruta P, Sahu JN, Anil Kumar P, Prabir G (2023). Current perspective of nano-engineered metal oxide based photocatalysts in advanced oxidation processes for degradation of organic pollutants in wastewater. Chem. Eng. Res. Des..

[5] Huang J (2020). Excellent visible light responsive photocatalytic behavior of N-doped TiO_2_ toward decontamination of organic pollutants. J. Hazard. Mater..

[6] Zheng X (2021). Unidirectional/bidirectional electron transfer at the Au/TiO_2_ interface operando tracked by SERS spectra from Au and TiO_2_. ACS Appl. Mater. Interfaces.

[7] Linsebigler AL, Lu G, Yates JT (1995). Photocatalysis on TiO_2_ surfaces: principles, mechanisms, and selected results. Chem. Rev..

[8] Kumar A (2021). Recent advances in plasmonic photocatalysis based on TiO_2_ and noble metal nanoparticles for energy conversion, environmental remediation, and organic synthesis. Small..

[9] Cai W (2022). TiO_2_ Thickness-dependent charge transfer effect in p-aminothiophenol molecules chemisorbed on TiO_2_/Ni substrates. Appl. Surf. Sci..

[10] Anxin J (2021). Aligned TiO_2_ nanorod arrays decorated with closely interconnected Au/Ag nanoparticles: Near-infrared SERS active sensor for monitoring of antibiotic molecules in water. Sensor Actuat. B: Chem..

[11] Shet A, Shetty KV (2015). Photocatalytic degradation of phenol using Ag core-TiO_2_ shell (Ag@TiO_2_) nanoparticles under UV light irradiation. Environ. Sci. Pollut. Res. Int..

[12] Huang Q (2017). Synthesis, characterization and application of TiO_2_/Ag recyclable SERS substrates†. Rsc Adv.

[13] Kumar S, Lodhi DK, Singh JP (2016). Highly sensitive multifunctional recyclable Ag–TiO_2_ nanorod SERS substrates for photocatalytic degradation and detection of dye molecules. RSC Adv..

[14] Qin S, Cai W, Tang X, Yang L (2014). Sensitively monitoring photodegradation process of organic dye molecules by surface-enhanced Raman spectroscopy based on Fe_3_O_4_@SiO_2_@TiO_2_@Ag particle. Analyst.

[15] Wang S (2020). Preferentially oriented Ag-TiO_2_ nanotube array film: An efficient visible-light-driven photocatalyst. J. Hazard. Mater..

[16] Li H (2020). Fabrication of pollutant-resistance SERS imprinted sensors based on SiO_2_@TiO_2_@Ag composites for selective detection of pyrethroids in water. J. Phys. Chem. Solids.

[17] Xiao Y (2009). The strategies of molecular architecture and modification of polyimide-based membranes for CO_2_ removal from natural gas—a review. Prog. Polym. Sci..

[18] Liaw D-J (2012). Advanced polyimide materials: Syntheses, physical properties and applications. Prog. Polym. Sci..

[19] Dong G (2019). TiO_2_ nanoshell@polyimide nanofiber membrane prepared via a surface-alkaline-etching and in-situ complexation-hydrolysis strategy for advanced and safe LIB separator. J. Membr. Sci..

[20] Nah C (2003). Characteristics of polyimide ultrafine fibers prepared through electrospinning. Polym Int.

[21] Hu Y (2019). Enhanced photocarrier separation in conjugated polymer engineered CdS for direct Z-scheme photocatalytic hydrogen evolution. Appl Catal B-Environ.

[22] Hu L (2019). Direct electrospinning method for the construction of Z-scheme TiO_2_/g-C_3_N_4_/RGO ternary heterojunction photocatalysts with remarkably ameliorated photocatalytic performance. Chinese J. Catal.

[23] Zhou J (2017). A (001) dominated conjugated polymer with high-performance of hydrogen evolution under solar light irradiation. Chem. Commun..

[24] Zhang Y (2020). Construction of TiO_2_/Ag_3_PO_4_ nanojunctions on carbon fiber cloth for photocatalytically removing various organic pollutants in static or flowing wastewater. J. Colloid Interface Sci..

[25] Xiong H (2019). Controllable synthesis of mesoporous TiO_2_ polymorphs with tunable crystal structure for enhanced photocatalytic H_2_ production. Adv. Energy Mater..

[26] Shi H (2017). Polyoxometalate/TiO_2_/Ag composite nanofibers with enhanced photocatalytic performance under visible light. Appl. Catal. B..

[27] Zhou JK (2008). Synthesis of self-organized polycrystalline F-doped TiO_2_ hollow microspheres and their photocatalytic activity under visible light. J. Phys. Chem. C..

[28] Zhiyuan C (2013). Inverse opal structured Ag/TiO_2_ plasmonic photocatalyst prepared by pulsed current deposition and its enhanced visible light photocatalytic activity. J. Mater. Chem. A..

[29] Guo Q, Li H, Zhang Q, Zhang Y (2018). Fabrication, characterization and mechanism of a novel Z-scheme Ag_3_PO_4_/NG/polyimide composite photocatalyst for microcystin-LR degradation. Appl Catal B: Environ..

[30] Meng P, Heng H, Sun Y, Liu X (2018). In situ polymerization synthesis of Z-scheme tungsten trioxide/polyimide photocatalyst with enhanced visible-light photocatalytic activity. Appl. Surf. Sci..

[31] Fu J (2017). Hierarchical porous O‐doped g‐C_3_N_4_ with enhanced photocatalytic CO_2_ reduction activity. Small.

[32] Gai L (2013). Controlled synthesis of nitrogen-doped binary and ternary TiO_2_ nanostructures with enhanced visible-light catalytic activity. J. Solid State Chem..

[33] Dong G (2020). Hierarchical mesoporous titania nanoshell encapsulated on polyimide nanofiber as flexible, highly reactive, energy saving and recyclable photocatalyst for water purification. J. Clean. Prod..

[34] Gyawali G (2013). Sonochemical synthesis of solar-light-driven Ag̊-PbMoO_4_ photocatalyst. J. Hazard. Mater..

[35] Xiang Q (2010). Au NAnoparticle Modified WO_3_ nanorods with their enhanced properties for photocatalysis and gas sensing. J. Phys. Chem. C.

[36] Linic S, Christopher P, Ingram DB (2011). Plasmonic-metal nanostructures for efficient conversion of solar to chemical energy. Nat. Mater..

[37] Chen SF (2010). Large scale photochemical synthesis of M@TiO_2_ nanocomposites (M = Ag, Pd, Au, Pt) and their optical properties, CO oxidation performance, and antibacterial effect. Nano Res..

[38] Wang M, Han J, Xiong H, Guo R (2015). Yolk@Shell Nanoarchitecture of Au@r-GO/TiO_2_ Hybrids as Powerful Visible Light Photocatalysts. Langmuir..

[39] Yang S (2020). Monitoring the charge-transfer process in a Nd-doped semiconductor based on photoluminescence and SERS technology. Light Sci. Appl..

[40] Leng WH, Zhang Z, Zhang JQ, Cao CN (2005). Investigation of the kinetics of a TiO_2_ photoelectrocatalytic reaction involving charge transfer and recombination through surface states by electrochemical impedance spectroscopy. J. Phys. Chem. B.

[41] Chen D (2013). Synthesis and Ag-loading-density-dependent photocatalytic activity of Ag@TiO_2_ hybrid nanocrystals. Appl. Surf. Sci..

[42] Chai B (2018). In-situ synthesis of WO_3_ nanoplates anchored on g-C_3_N_4_ Z-scheme photocatalysts for significantly enhanced photocatalytic activity. Appl. Surf. Sci..

[43] Zhu B (2017). Fabrication and photocatalytic activity enhanced mechanism of direct Z-scheme g-C_3_N_4_/Ag_2_WO_4_ photocatalyst. Appl. Surf. Sci..

[44] Kumar SG, Rao KSRK (2017). Comparison of modification strategies towards enhanced charge carrier separation and photocatalytic degradation activity of metal oxide semiconductors (TiO_2_, WO_3_ and ZnO). Appl. Surf. Sci..

[45] Wang Y (2020). Photocatalytic activity of N-TiO_2_/O-doped N vacancy g-C_3_N_4_ and the intermediates toxicity evaluation under tetracycline hydrochloride and Cr (VI) coexistence environment. Appl. Catal. B.

[46] Chen Y (2011). Naturally occurring sphalerite as a novel cost-effective photocatalyst for bacterial disinfection under visible light. Environ. Sci. Technol..

[47] Yu F (2014). The preparation and properties of a g-C_3_N_4_/AgBr nanocomposite photocatalyst based on protonation pretreatment. New J. Chem..

[48] Cui K (2018). Para-aminothiophenol radical reaction-functionalized gold nanoprobe for one-to-all detection of five reactive oxygen species in vivo. Anal. Chem..

[49] Hu M (2020). Self-cleaning semiconductor heterojunction substrate: ultrasensitive detection and photocatalytic degradation of organic pollutants for environmental remediation. Microsyst. Nanoeng..

[50] Wu T (2021). Self-sustainable and recyclable ternary Au@Cu_2_O-Ag nanocomposites: application in ultrasensitive SERS detection and highly efficient photocatalysis of organic dyes under visible light. Microsyst. Nanoeng..

